# Exit from Competence for Genetic Transformation in *Streptococcus pneumoniae* Is Regulated at Multiple Levels

**DOI:** 10.1371/journal.pone.0064197

**Published:** 2013-05-22

**Authors:** Liming Weng, Andrew Piotrowski, Donald A. Morrison

**Affiliations:** Department of Biological Sciences, University of Illinois at Chicago, Chicago, Illinois, United States of America; University of Florida, United States of America

## Abstract

Development of natural competence in *S. pneumoniae* entails coordinated expression of two sets of genes. Early gene expression depends on ComE, a response regulator activated by the pheromone CSP (**C**ompetence-**S**timulating-**P**eptide). Subsequently, an early gene product (the alternative sigma factor ComX) activates expression of late genes, establishing the competent state. Expression of both sets of genes is transient, rapidly shut off by a mechanism that depends on the late gene, *dp*r*A*. It has been thought that the rapid shutoff of late gene expression is the combined result of auto-inhibition of ComE and the instability of ComX. However, this explanation seems incomplete, because of evidence for ComX-dependent repressor(s) that might also be important for shutting off the response to CSP and identifying *dprA* as such a gene. We screened individual late gene mutants to investigate further the roles of ComX-dependent genes in competence termination. A Δ*dprA* mutant displayed a prolonged late gene expression pattern, whereas mutants lacking *cbpD, cibABC, cglEFG, coiA, ssbB, celAB, cclA, cglABCD, cflAB, or radA*, exhibited a wild-type temporal expression pattern. Thus, no other gene than *dprA* was found to be involved in shutoff. DprA limits the amounts of ComX and another early gene product, ComW, by restriction of early gene expression rather than by promoting proteolysis. To ask if DprA also affects late gene expression, we decoupled late gene expression from early gene regulation. Because DprA did not limit ComX activity under these conditions, we also conclude that ComX activity is limited by another mechanism not involving DprA.

## Introduction

*Streptococcus pneumoniae*, a gram positive bacterium inhabiting the human upper respiratory tract, is an opportunistic pathogen which causes many kinds of infection, such as pneumonia, meningitis and otitis. One of the most remarkable features of this microbe is its ability of natural genetic transformation, or competence, discovery of which led to the identification of DNA as the genetic material [1]. In laboratory cultures of *S. pneumoniae*, an outburst of competence occurs during the mid-log phase, emerging and shutting off rapidly, within about 30 minutes. Recent research has uncovered several aspects of the regulatory process by which virtually all cells of an unsynchronized pneumococcal culture shift, in concert, from an incompetent state to a fully competent state [2]. It starts with the synthesis of a polypeptide ComC, which is exported through a cell membrane transporter ComAB, and processed to a 17-amino-acid peptide, named CSP (competence stimulating peptide). Acting as a pheromone, CSP binds ComD and activates the two component system: histine kinase ComD and response regulator ComE. Phosphorylated ComE is thought to act as a transcriptional activator, recognizing an imperfect direct repeat in front of the promoters of several genes, termed early genes. Among these are *comAB* and *comCDE*, which act to produce more CSP, and *comX* and *comW*, whose products together turn on expression of the set of genes termed late genes. ComX acts as an alternative sigma factor recognizing a promoter sequence termed the "cinbox” or “combox". Finally, some late genes up-regulated by ComX participate in a variety of functions related to transformation, including DNA uptake and processing, recombination, fratricide, and immunity to fratricide, while others have roles that remain to be discovered, but are not required for transformation. While it is clear that the auto-catalytic peptide pheromone CSP serves to coordinate development of competence among the cells of a culture, the signals or stresses that trigger the developmental cycle are only beginning to be discovered [3]–[5].

During the period of maximal competence, also termed the X state [3], transcription is dominated by an excess of the alternative sigma factor, ComX, which is otherwise entirely absent from the cell. To escape from this state, key connections in the circuit must be interrupted decisively. A dramatic temporal pattern of mRNA accumulation and loss in response to an acute dose of the CSP signal attests to the coordination and strength of these effects [6]–[8]. A brief period of early gene expression is followed by a brief period of late gene expression and a somewhat longer period of competence reflecting the activities of the accumulated late gene products.

The lag in expression of the late genes is explained by the role of the early gene product ComX as an alternative sigma factor driving expression of late genes from specific non-canonical promoters. However, as rapidly as this chain of responses to CSP is established, its effects are nearly as rapidly reversed [6], [7], [9], [10], leading to the remarkably transient nature of competence. Early gene mRNA quickly accumulates, increasing at least 100-fold between 2 and 10 min after a sudden increase in CSP level, but these messages then disappear just as quickly, leaving less than 10% of maximal levels in the cell by 15 min. As no specifically targeted anti-mRNA mechanism is known, this suggests that ComE-directed transcription stops abruptly by 10 min. While the ComE protein itself is stable [11], a prime candidate for the cause of this change is a change in the amount of phosphorylated ComE (ComEP). Transcripts of late genes follow a delayed but similar temporal course, peaking at 13 min and largely disappearing by 17 min, implying as well that ComX activity is largely dissipated by 13 min. Although labile, ComX protein is present well past 20 min, suggesting that some inhibition of ComX activity occurs before it physically decays. A further indication of an antagonist of ComX activity was recently provided: while mutation of ClpP stabilized ComX, it failed to prolong its transcriptional activity, detected either as prolonged transformation or as transcription of late genes [12]. Thus, there appear to be at least two targets of competence shutoff mechanisms, the activity of ComEP (which might be inactivated by a phosphatase) and the activity of ComX.

Martin et al [13] reported that the ComE R120S mutant exhibits a much delayed shutoff of competence, with transformation continuing past 90 min, much longer than the 20-min period of competence in wild type cultures. This behavior indicates that ComE activity is an important determinant of the exit from competence and that cells in a competent culture can support an extended period of transformation. Indeed, Claverys and Håvarstein [2] proposed that ComE itself acts to shut off its own activity when accumulated to a high enough level. Thus, it has been thought that exit from competence might be a two-step process involving, first, dephosphorylation or other inactivation of the stable regulator, ComE, followed by degradation of the unstable proteins, ComX and ComW [2], [14]. More recently the case for the importance of the second, proteolytic, step was clouded when it was observed that competence still shuts off in a strain where ComX and ComW are stabilized by inactivation of cognate proteases [12]. Furthermore, competence induced by ectopic expression of *comX* and *comW*, without participation of other early genes, also follows a course leading to rapid shutoff [15]. These results together indicate that there is at least one factor shutting off competence by targeting ComX directly, independent of proteolysis of ComX and independent of any regulatory effects on expression of early genes.

Additional clues to the mechanism by which competence is terminated are provided by the phenotypes of *comX* and *dprA* mutants. While *comX* mutants do not transform, or become competent by the criterion of expression of late genes, they do respond to CSP, with over-expression of ComX [9], [16] and other early genes [7]. The temporal pattern of this response is remarkable: it is not rapidly reversed, as in wild type; instead the induced early gene expression continues for generations [16]. This simple result immediately suggests that one or more late gene products may be important for the reversal of the CSP response, although another logical possibility is that ComX itself acts as such a repressor. A remarkably similar regulatory phenotype was described for mutants defective in the late gene, *dprA*. Bergé reported that the *dprA* mutation blocked transformation (later explained by roles of DprA in stabilization or recombination of donor DNA fragments), but also that it causes an exacerbated (∼60 min) growth arrest upon CSP treatment, while simultaneously permitting continued expression of the *comCDE* early competence operon [17].

**Figure 1:**
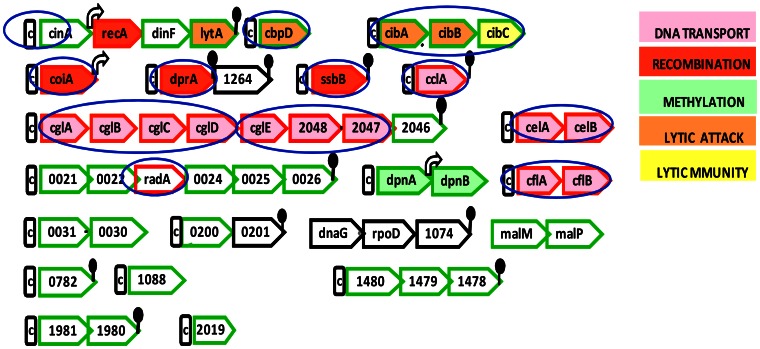
Organization of late genes of the *S.* *pneumoniae* competence regulon. Internal deletion mutants studied in this paper are indicated by blue ovals. C represents the cinbox promoter, TACGAATA. Fill colors symbolize the functions of proteins, as indicated by the key on the right. Bent arrow and lollipop stand for constitutive promoter and terminator respectively. Red borders indicate genes required for transformation; green borders indicate genes not required for transformation. Black borders indicate genes whose importance for transformation has not been directly determined. Genes described by numbers (ORF numbers in TIGR4) are those whose functions in competence are unknown. The ORF numbers for named late genes are: *cinA*(sp1941), *cbpD*(sp2201), *cibA*(sp0125), *coiA*(sp0978), *dprA*(sp1266), *ssbB*(sp1908), *cclA*(sp1808), *cglA*(sp2053), *celA*(sp0054), *cflA*(sp2208). The late gene clusters and associated cinbox are as in Peterson et al [9], but without cases of apparent read-through transcription identified in [34].

To search more broadly for late gene(s) implicated in reversal of the response to CSP, Peterson et al [7] examined the competence kinetics of many mutants defective in genes that were induced in competent cells but not required for transformation (green genes in Figure 1). None of the transformable mutant strains tested displayed an extended period of competence. Mirouze et al. subsequently demonstrated that it was possible to complement the regulatory defect of a *comX* mutant in escape from the CSP response, restoring transient expression of the *comCDE* operon, by ectopic *comX*-independent expression of *dprA,* under control of an early class promoter [18]. They proposed that the relevant activity of DprA might be either to promote de-phosphorylation of ComE or to block phospho-transfer to ComE by ComD, either of which would be expected to cause a broad effect on all early genes in addition to the effect they observed on the *comCDE* operon.

Although DprA thus seems necessary for shutoff of the (early) *comCDE* operon and sufficient for ComX-independent shutoff of *comCDE* transcription, its regulatory target remains uncertain, and it is unknown whether additional late genes might contribute as well to exit from the X state. To identify possible additional pieces of this puzzle, we looked further among the late genes for products that act to promote the exit from competence. Although the late genes outlined in red in Fig. 1 (*cclA, celA, cflA, cglA, coiA, radA, recA,* and *ssbB*) are required for transformation and have critical roles in DNA transport, processing, or recombination, they had not been examined for possible roles in competence termination, perhaps because of the defective transformation in such mutants. This impediment could be circumvented with an indirect method, simply by monitoring shutoff of competence gene expression, instead of decay of transformability *per se* as an indicator of exit from the competent state. Since the late gene expression pattern is itself a reliable indicator of competence kinetics [17], [19] and late gene products are the effectors of transformation, the expression pattern of a late gene revealed by a simple LacZ assay in a ComX**^+^** background will best reflect competence development and persistence, even in non-transformable mutants. Using this strategy, with a *lacZ* transcriptional reporter at the *ssbB* late gene, we examined mutants defective in additional late genes that are required for transformation. The mutant lacking *dprA* displayed, as expected, a prolonged late gene expression pattern, whereas mutants lacking *cbpD, cibABC, cglEFG, coiA, ssbB, celAB, cclA, cglABCD, cflAB, or radA*, exhibited the wild-type pattern of rapid extinction of late gene expression. We further report that closer examination of the effects of DprA on competence regulation revealed that DprA interacts with ComE to control early gene expression but does not affect proteolysis of labile early gene products, while a second, *dprA*-independent and non-proteolytic, mechanism targets ComX activity during escape from the X-state.

## Materials and Methods

### Bacterial Strains, Oligonucleotides, Plasmids, and Culture Media

All pneumococcal strains used in this study are described in Table 1. The transformation-proficient strains are the derivatives of the Rx1 strain [20] which carries a mis-sense mutation in *cps3D* [21]. CP2000 carries a deletion of all capsular polysaccharide synthesis genes between *aliA* and *dexB*, respectively. CPM7 is a derivative of CP1250 with the *lacZ* reporter plasmid pEVP3 inserted in the *ssbB* locus, with a truncated *ssbB* upstream of the insert and an intact *ssbB* downstream. All the strains were grown in the casein hydrolysate/tryptone medium, CAT [16], [22]. Antibiotics were used at the following selective concentrations: erythromycin (Em), 0.05 µg/ml; streptomycin (Sm), 100 µg/ml; kanamycin (Kan), 200 µg/ml; and tetracycline (Tet), 0.25 µg/ml. CSP1 [23] was obtained as a custom peptide from Mimitopes LLC (Raleigh). Synthetic oligonucleotide primers, purchased from Eurofins MWG Operon (Huntsville), are listed in Table 2. *E. coli* strains were grown to late exponential phase in LB media [24], and stored with 20% sterile glycerol. For use, the frozen stocks were streaked onto LB agar plates with appropriate antibiotics, and a single colony was then inoculated into fresh medium. Ampicillin was used 0.1 µg/ml for selection of *E. coli*. Yeast strains s were grown in YPD or SD synthetic media [25], and stored with sterile glycerol at 20% final concentration. For use, frozen stocks were streaked onto YPD agar plate, and a single colony was then inoculated into fresh YPD or synthetic selective media. 1 mM or 3 mM 3AT (3-Amino-1,2,4-triazole) was used to select against histidine production. Plasmids used in this study are listed in Table 3.

**Table 1:** Bacterial strains used in this study.

Strain	Description	Source (a) or reference
Streptococcus. pneumoniae		
CPM7	CP1250, but ssbB^−^::lacZ::ssbB^+^; SsbB^+^ Sm^R^ Cm^R^	[22]
CP1250	Rx, but cps3D hex cps3D malM511 str-1 bgl-1; Hex^−^ Mal^−^ Sm^R^ Bga^−^	[29]
CP1275	CP1250, but ΔcbpD::PcKan; Kan^R^	[7]
CP1279	CP1250, but ΔcibABC::PcKan; Kan^R^	[7]
CP1333	CP1250, but ΔcglEFG::PcKan; Kan^R^	[35]
CP1344	CP1250, but ΔclpC::P_C_Tet; Tet^R^	[12]
CP1359	CP1250, but ΔclpP::PcTet; Tet^R^	[12]
CP1389	CP1250, but ΔdprA::PcKan; Kan^R^	[36]
CP1415	CP1250, but Δ*comA*::PcErm; Em^R^	[37]
CP1500	Rx, hex nov-r1, byr-r, ery-r1,ery-r2, str-1;Nov^R^ Em^R^ Sm^R^,	[20]
CP1793	CP1250, but ΔcoiA::PcKan; Kan^R^	[36]
CP1851	CP1250, but ΔclpE::PcErm; Em^R^	[12]
CP1862	CP1250, but ΔcelAB::PcKan; Kan^R^	This work
CP1863	CP1250, but ΔcclA::PcKan; Kan^R^	This work
CP1868	CP1250, but ΔcglABCD::PcKan; Kan^R^	This work
CP1869	CP1250, but ΔcflAB::PcKan; Kan^R^	This work
CP1890	CP1250, but ssbB^−^::lacZ::ssbB^+^, ΔclpP::PcTet; Cm^R^ Tet^R^	CPM7×CP1359
CP1894	CP1250, but ssbB^−^::lacZ::ssbB^+^, ΔdprA::PcKan; Cm^R^ Kan^R^	CPM7×CP1389
CP1895	CP1250, but ssbB^−^::lacZ::ssbB^+^, ΔclpP, ΔdprA; Cm^R^ Kan^R^ Tet^R^	CP1890×CP1389
CP1896	CP1250, but aga::comX::comW::PcKan; Kan^R^	This work
CP1961	CP2000, but aga::comX::comW, ssbB^−^::lacZ::ssbB^+^; Cm^R^ Kan^R^	CP1896×CP2000×CPM7
CP1962	CP1961, but ΔclpE::PcErm; Em^R^	CP1961×CP1851
CP1963	CP1961, but ΔclpC::P_C_Tet; Tet^R^	CP1961×CP1344
CP1902	CP1961, but ΔclpC::P_C_Tet, ΔclpE::PcErm; Tet^R^ Em^R^	CP1962×CP1344
CP1932	CP1902, but ΔdprA::PcKan; Kan^R^	CP1902×CP1389
CP2000	CP1250, but Δcps; Hex^−^ Mal^−^ Cps^−^ Sm^R^ Bga^−^	This work
CP2108	CP2000, but ssbB^−^::lacZ::ssbB^+^, ΔcomA::PcErm; Sm^R^ Cm^R^ Em^R^	CP2000×CPM7×CP1415
CP2109	CP2108, but ΔcbpD::PcKan; Kan^R^	CP2108×CP1275
CP2110	CP2108, but ΔcibABC::PcKan; Kan^R^	CP2108×CP1279
CP2111	CP2108, but ΔcoiA::PcKan; Kan^R^	CP2108×CP1793
CP2112	CP2108, but ΔcglEFG::PcKan; Kan^R^	CP2108×CP1333
CP2113	CP2108, but ΔdprA::PcKan; Kan^R^	CP2108×CP1389
CP2114	CP2108, but ΔcclA::PcKan; Kan^R^	CP2108×CP1863
CP2115	CP2108, but ΔcflAB::PcKan; Kan^R^	CP2108×CP1869
CP2116	CP2108, but ΔcelAB::PcKan; Kan^R^	CP2108×CP1862
CP2117	CP2108, but ΔcglABCD::PcKan; Kan^R^	CP2108×CP1868
CP2118	CP1250, but ΔradA::PcSpc; Spc^R^	This work
CP2119	CP2108, but ΔradA::PcSpc; Spc^R^	CP2108×CP2118
CP2125	CP2108, but ΔclpP::PcTet; Tet^R^	CP2108×CP1359
CP2126	CP2125, but ΔcbpD::PcKan; Kan^R^	CP2125×CP1275
CP2127	CP2125, but ΔcibABC::PcKan; Kan^R^	CP2125×CP1279
CP2128	CP2125, but ΔcglEFG ::PcKan; Kan^R^	CP2125×CP1333
CP2129	CP2125, but ΔdprA::PcKan; Kan^R^	CP2125×CP1389
CP2130	CP2125, but ΔcoiA::PcKan; Kan^R^	CP2125×CP1793
CP2131	CP2125, but ΔcelAB::PcKan; Kan^R^	CP2125×CP1862
CP2132	CP2125, but ΔcclA::PcKan; Kan^R^	CP2125×CP1863
CP2133	CP2125, but ΔcglABCD::PcKan; Kan^R^	CP2125×CP1868
CP2134	CP2125, but ΔcflAB::PcKan; Kan^R^	CP2125×CP1869
CP2135	CP2125, but ΔradA::PcSpc; Spc^R^	CP2125×CP2119
CP2139	CP2108, but ΔssbB::PcKan; Kan^R^	This work
CP2140	CP2125, but ΔssbB::PcKan; Kan^R^	This work
CP2143	CP2108, but ΔPc-cinA::PcKan; Kan^R^	This work
CP2144	CP2125, but ΔPc-cinA::PcKan; Kan^R^	This work
Escherichia coli		
DH5α	F-recA1, endA1 hsdR17 phoA supE44 thi-1 gyrA96	Invitrogen
Saccharomyces cerevisiae		
NSY468	MATa, trp1-901, leu2-3, l 12, ura3-52, his3-200, gal4Δ, gal80ΔGAL2-ADE2, LYS2::GAL1-HIS3, met2::GAL7-lacZ	[38]
NSY752	MATα, trp1-901, leu2-3, l 12, ura3-52, his3-200, gal4Δ, gal80ΔGAL2-ADE2, LYS2::GAL1-HIS3, met2::GAL7-lacZ	[38]

a Crosses are indicated as recipient X donor genomic DNA.

**Table 2:** Primers used for strain construction.

Primer	Sequence (5′-3′)	Size (kb)		Construct
**Kan**				
DAM303	AAGGGCCCGTTTGATTTTTAATG	0.8	Kan^R^ marker	
DAM304	AGGATCCATCGATACAAATTCCTC			
Δ**cps3**				
TTM01	ATCATGACCTCCCTCGTATTGT	0.8	Upstreamfragment	
TTM02	CGCGGATCCTTAATAGTGGGAATTTG			
DAM823	CGCGGATCCTTGGAGTTAGAATAGGGCA	1.5	Downstreamfragment	
DAM827	GCCTCATCACCAGCCTCAGTAAC			
Δ**ssbB**				
DAM934	GCATGGGCCCTGAAGAAAGCAGACAAGTAAGC	0.7	Upstreamfragment	
DAM935	GGCCTATCTGACAATTCCTG			
BVD 104	ATGGATCCTGCCATTTTAAGAATTAAAAAGTC	1.0	Downstreamfragment	
BVD 105	GACTCTTCGATGGTGATGACACCGTCTTTG			
Δ**coiA**				
BVD26	AAACGGGAGTCTATCAAACGTCGTGAGCAA	0.8	Upstreamfragment	
BVD27	ATGGATCCTGAATTCCCTCCTTTTCTATATCAT			
BVD28	ATGGGCCCGAATAGAAAGGATGGAGGAATCTAA	1.5	Downstreamfragment	
BVD29	GTAGACATCGTACATCTTGAGATCTGAAAT			
Δ**cibABC**				
DAM305	CAAGGACTGACTAGGTAAACAGC	0.7	Upstreamfragment	
DAM306	GCTAGGATCCGAGGGCACTCTTGTCTGG			
DAM307	ACGAGGGCCCGATAGCAAAAGCAAATAA	0.9	Downstreamfragment	
DAM308	CAAGAGGCCGTGTTCTTCGAG			
Δ**cbpD**				
DAM313	AGCTTTCTCGTGGTGTAGAACAAC	1.6	Upstreamfragment	
DAM314	ACGAGGATCCGATCCATTTCCTCTGGAATA			
DAM315	AGCAGGGCCCAGGTCTCTGGTAAGTGGTAT	0.8	Downstreamfragment	
DAM316	CTCTCAAGGTCGCCCAGCTATG			
Δ**cglEFG**				
DAM419	CTGTAATTGAGCCTCCGTTACCAATATG	1.2	Upstream fragment	
DAM420	ATGGATCCGAGTCTGGTTGCTATGATTAGTCT			
DAM421	ATGGGCCCTTAGCTACCCTCAAGACTTCTTC	1.5	Downstream fragment	
DAM422	TTGTGCAGACCTACTTGACAGCCTATTATG			
Δ**dprA**				
DAM563	GATAGAGGCGATAAGCATGGCACATAGTAA	1.0	Upstream fragment	
DAM564	ATGGGCCC-TGCCATCATTTGATTCAAGAAG			
DAM565	GGATCC-ATAACGGCTGGATTACGGCAACCT	2.0	Downstream fragment	
DAM566	GATTGGGAACTCGCTTGCGTCCTATGACTGA			
Δ**celA**				
DAM659	CTAATTCTGGAGCAGGCGGCCATGTG	1.1	Upstream fragment	
DAM660	CGCGGATCC-TTTCAACTGCTTATTTATTTGC			
DAM661	ACGTGGGCCC-GGAAGGATAAATGTTGTAGATTAG	0.8	Downstream fragment	
DAM662	TGAGCCAGCATTTGGCCTGACTGAG			
Δ**cclA**				
DAM663	TGTTGAGTGGCGACGATAAATAAGG	1.1	Upstream fragment	
DAM664	CGCGGATCC-TAGTATAATGGAGAAACATAGATAAG			
DAM665	ACGTGGGCCC-TTGTTTGATAAAGTCCAATTTC	1.2	Downstream fragment	
DAM666	AACAAGCCATTTGGCAGTTTGAGTC			
Δ**cglABCD**				
DAM680	TGCAGCGTAGCCATTATTGGTTCAG	1.2	Upstreamfragment	
DAM679	CGCGGATCC-TCCTCACCTATACTATTCGCAAAG			
DAM682	ACGTGGGCCC-TGATTTTACTGGAAGCAGTAGTC	1.0	Downstreamfragment	
DAM681	ATCCGTACGAACCCTCGTCACTAAG			
Δ**cflAB**				
DAM684	TTCAATCATGCTAAGGGCAATACGG	1.2	Upstreamfragment	
DAM683	CGCGGATCC-AATCATGGAATTTAGGACAATTAAAG			
DAM686	ACGTGGGCCC-TCATAAAAACAAAAATGTTTAG	1.0	Downstreamfragment	
DAM685	ACGTGGGCCCTCATAAAAACAAAAATGTTTAG			
Δ**P_com_-cinA**				
DAM936	GCATGGGCCCCGCAGGAATTTTCCTACGATTG	1.0	Upstreamfragment	
DAM937	CAAGGGACAGAAACCTTAGC			
DAM938	GTCAGGATCCGAGTGGCAGGACCAGATAG	1.2	Downstreamfragment	
DAM939	GGTGCTCTGCCAAGTATTTC			
**aga::comXcomW::P_c_Kan**				
DAM786	AAACTGGGTGGAAGTCTAGAAAGTC	1.4	aga::comX	
PL82	CGCGGATCCTGACTTACTAATGGGTACG			
DAM791	ATCGAATTCGGATCCGTTTGATTTTTAATGG	0.4	P_c_Kan:rafE	
DAM793	AACATCGGTATAGCCAGCACCTTCC			
DAM794	ATCGGATCCAAAAAAGAAAAGGAGTATTTGA	2.0	comW	
DAM790	CTAGAATTCCTCAACAAGAAATAAACCCCC			
**pACT2 or pGBDUC2 inserts**				
DAM967	gtaggatccAGTTATTTATGAAAATCACAAACTATGAAATCT	0.9	pGBDUC2::dprA	
DAM968	gcatgtcgacTTAAAATTCAAATTCCGCAAGAACATC			
DAM969	cagtggatccAAAGAGTAATGGATTTATTTGGATTTG	1.3	pACT2::comD	
DAM970	gcatctcgagCTTTCATTCAAATTCCCTCTTAAATCTA			
DAM971	gtacggatccGAATGAAAGTTTTAATTTTAGAAGATGTTATTG	0.8	pACT2::comE	
DAM972	gcatctcgagTCAATCACTTTTGAGATTTTTTCTCTAA			
DAM973	gtacggatccAGGGGAAAATTATGATTAAAGAATTGTAT	0.5	pACT2::comX	
DAM974	gcatctcgagCTAATGGGTACGGATAGTAAACTC			
DAM975	gtcaggatccTTATGTTACAAAAAATTTATGAGCAGATG	0.3	pACT2::comW	
DAM976	gcatctcgagTACTAAAATTACCTCAACAAGAAATAAAC			
DAM989	gactggatccGAATGGCGAAAAAACCAAAAAAATTA	1.2	pACT2::recA	
DAM990	gtcactcgagCAGCTTATTCTTCAATTTCGATTTCA			

**Table 3:** Plasmids used in this studies.

Plasmid	Description	Source
pACT2	shuttle vector for yeast 2-hybrid, carrying Gal4 activating domain (AD)	Clontech Labs, Inc.
pGBDUC2	shuttle vector for yeast 2-hybrid, carrying Gal4 DNA binding domain (BD)	[38]
pACT2-*comD*	pACT2 derivative, carrying AD-*comD* fusion	This work
pACT2-*comE*	pACT2 derivative, carrying AD-*comE* fusion	This work
pACT2-*comX*	pACT2 derivative, carrying AD-*comX* fusion	This work
pACT2-*comW*	pACT2 derivative, carrying AD-*comW* fusion	This work
pACT2-*dprA*	pACT2 derivative, carrying AD-*dprA* fusion	This work
pACT2-*recA*	pACT2 derivative, carrying AD-*recA* fusion	This work
pGBDUC2-*dprA*	pGBDUC2 derivative, carrying BD-*dprA* fusion	This work

#### Transformation

For *S. pneumoniae*, cultures grown in CAT to OD550 = 0.03 were exposed to DNA (0.1 µg/ml) for 45 min after the treatment with CSP (0.25 µg/ml), CaCl_2_ (0.5 mM), and bovine serum album (0.04%). Transformants were selected in CAT solidified with 1.5% agar containing appropriate antibiotics. For *E. coli*, 60 µl desalted, electro-transformable cells in 10% glycerol were mixed with 3 µl plasmid DNA (∼100 ng) and electroporated with voltage 2.0 kV, resistance 200 Ω and capacitance 25 µF. After recovering in 1 ml LB broth medium at 37°C for one hour, the cell culture was spun down and plated. For yeast, 1 ml OD600>5 haploid cells were spun down and resuspended in 0.5 ml PEGLET buffer (40% PEG, 0.1 M LiAC, 10 mM EDTA, 0.1 M Tris-HCl, pH 7.5). About 100 µg ssDNA (salmon sperm DNA, Sigma) plus 0.5∼1 µg plasmid DNA were added to the resuspended cells. The cell culture was seated on the bench at room temperature overnight. Next day, the cells were spun down again and plated.

#### Creation of strain CP2000

To create the capsule-less strain CP2000 by use of the Janus replacement cassette [26], CP1250 was transformed with genomic DNA from strain R6J (R6S but *cps*::*kan-rpsL* ^+^) [27], using kanamycin for selection. One transformant with the correct structure was retained as strain CP1999. CP1999 was transformed with a donor DNA created by the ligation of two *Bam*HI digested PCR fragments. The first fragment was created by amplifying CP1500 genomic DNA with primers TTM01 and TTM02; the second fragment was created by amplifying the same genomic DNA with primers DAM823 and DAM827. A Sm^R^ transformant with the correct structure, removing genes between *dexB* and *aliA*, was retained as CP2000, after verification by PCR and sequencing of the new junction/deletion.

#### Construction of parental strains CP2108 and CP2125

The parent strain CP2108 was made by the crosses among three strains: CP2000, CPM7, and CP1415. CP2000 (*Δcps*), was transformed with CPM7 genomic DNA; a Cm^R^ transformant with pEVP3 inserted at *ssbB* and exhibiting CSP-dependent β-galactosidase activity was named CP2107. This new strain was further transformed with CP1415 genomic DNA, to incorporate a disrupted *comA* gene. The disrupted *comA* gene in an Em^R^ transformant was confirmed by PCR and the strain was named CP2108. Then CP2108 was transformed with CP1359 (Δ*clpP*::PcTet) genomic DNA to make CP2125.

#### Creation of ectopic *comXcomW* strain, CP1896

To create strain CP1896 (*aga::comX::comW::PcKan*), two fragments were amplified from CP1372 DNA: one contained part of *aga* and a complete *comX* (primers DAM786 and PL82); the second contained a Kn^R^ cassette and part of *rafE* (primers DAM791 and DAM792). A third fragment containing *comW* was amplified from CP1500 using DAM794 and DAM790. After digestion by *Bam*HI (Fermentas), and/or by *Eco*RI (Fermentas), the three fragments were purified, ligated, and used directly as donor for transforming strain CP1250 as described above. One Kn^R^ transformant was retained as strain CP1896 after sequencing the insert, which exactly matched the predicted sequence.

#### Preparation of late gene mutants

Late gene mutants used in this paper were obtained by transforming the parent strains (CP2108 and CP2125) with either the genomic DNA of strains containing the corresponding late gene disruption (*ΔcbpD, ΔcibABC, ΔcglEFG, ΔcoiA, ΔdprA,* and *ΔradA*), or with donor DNA synthesized by molecular cloning (Δ*P_c_cinA, ΔcelAB, ΔcclA, ΔcglABCD, ΔcflAB, and ΔssbB).* For synthesizing donor DNA, two pairs of primers were used to amplify the upstream and downstream sequences individually. Then, the two sequences were ligated with a Kan^R^ cassette to make up the donor DNA for transformation.

#### Induction of competence using CSP

Cultures of pneumococcus were started by inoculating 200 ml complete CAT medium plus 10 mM HCl with 1/100 volume of a frozen stock of cells (OD_550_ 0.1). At the first visible turbidity during growth at 37°C, 10 ml was transferred to a 18 mm by 150 mm tube for monitoring optical density. When the culture reached an OD_550_ of about 0.06, it was induced to competence with CaCl_2_ (to 0.5 mM), BSA (to 0.002%), and CSP (to 250 ng/ml). Samples were taken periodically from the culture for various analyses as described in subsequent sections.

#### Induction of competence using raffinose

Cultures of pneumococcus were started by inoculating 200 ml complete CAT medium plus 10 mM HCl with 1/100 volume of a frozen stock of cells (OD_550_ 0.1). At the first visible turbidity during growth at 37°C, 10 ml was transferred to a 18 mm by 150 mm tube for monitoring optical density. When the culture reached an OD_550_ of about 0.05, the culture was transferred to 30°C. When the culture reached an OD_550_ of about 0.1, it was induced to competence with CaCl_2_ (to 0.5 mM), BSA (to 0.002%), and raffinose (to 0.1% w/v). Samples were taken periodically from the culture for various analyses as described in subsequent sections.

#### Sampling for western analysis

For Western blot analyses 1.8-ml samples were withdrawn from the culture, chilled rapidly on dry ice, without freezing, and then kept at 4°C until harvesting by centrifugation (10,000×g, 2 min, 4°C). After each cell pellet was resuspended with 35 µl loading buffer (50 mM Tris-HCl (pH 6.8), 2% SDS, 0.1% bromophenol blue, 10% glycerol, and 100 mM dithiothreitol) and heated at 95°C for 10 min, 15 µl of the lysate was loaded into one lane of an SDS-PAGE gel.

#### SDS-PAGE

SDS-PAGE was done as described previously [24] using the Bio-Rad Mini-Protean II gel apparatus. Each gel was a 15-well, 1.5-mm-thick discontinuous gel composed of a 5% stacking gel and a 15% resolving gel prepared according to the manufacturer’s recommendations. Protein samples were prepared by mixing with one volume of loading buffer (100 mM Tris-HCl (pH 6.8), 4% SDS, 0.2% bromophenol blue, 20% glycerol, and 200 mM dithiothreitol) and heating at 95°C for 10 minutes. The gels were run in 25 mM Tris, 250 mM glycine, 0.1% SDS, pH 8.0 at 65 V until the dye reached the resolving gel, then at 120 V until the dye reached the bottom of the gel.

#### Western analysis

For Western analysis, SDS-PAGE gels were run as described above, washed 3 times for 5 min with 100 ml deionized water, and equilibrated for 15 min in transfer buffer (25 mM Tris, 192 mM glycine, 10% (v/v) methanol, pH 8.0). The proteins were trans-blotted from the gel to a PVDF membrane (Immobilon P^sq^, Millipore) in transfer buffer for 2 hr at 36 V at 4°C. The membrane was then blocked overnight at 4°C in TBS-T (20 mM Tris-HCl (pH 7.6), 137 mM NaCl, 1% (v/v) Tween-20) with 5% nonfat dry milk. The membrane was incubated for 90 min at room temperature with primary antibody, specific for ComW or ComX, diluted by 1∶3000 in 5 ml TBS-T with 1% nonfat dry milk in a small plastic pouch. After washing 3 times with 100 ml TBS-T, then incubating for 1 hr at room temperature with secondary antibody (Anti-Rabbit IgG conjugated to HRP, Amersham) diluted 1∶20 000 in TBS-T with 1% nonfat dry milk and then washing 3 times with 100 ml TBS-T, the position of secondary antibody on the membrane was detected using an ECL substrate (ECL Plus, Amersham) and either Hyblot CL film (Denville Scientific) or the Alpha Imager CCD Camera (Alpha Innotech). Typical exposure times were 1 to 5 min for the film and 5 to 15 min for the CCD camera. Quantification was done by spot densitometry using AlphaEaseFC (Alpha Innotech).

#### β-galactosidase assay

For measurement of β-galactosidase activity, a 0.4-ml sample of liquid culture was chilled on ice. After adding 100 µl 5×Z buffer (300 mM Na_2_HPO_4_, 200 mM NaH_2_PO_4_, 50 mM KCl, 5 mM MgSO_4_, 250 mM β-mercaptoethanol, 0.5% TritonX-100) and incubation at 37 C for 10 min to lyse the cells, 150 µl of the resulting lysate was combined with 50 µl of o-nitrophenyl-β-D-galactopyranoside solution (4 mg/ml o-nitrophenyl-β-D-galactopyranoside, 60 mM Na_2_HPO_4_, 40 mM NaH_2_PO_4_) in a 96-well microplate. The plate was incubated at 37 C, and absorbance at 420 nm was read every 10 min for 90 min in Spectra Max M2 of Molecular Devices. The initial slope of the absorbance curve was used to calculate LacZ activity, reported in Miller units.

#### α-galactosidase assay

For α-galactosidase activity measurement, a 0.4-ml culture sample was added to 0.1 ml of 5×lysis buffer (405 mM Na_2_HPO_4_, 95 mM NaH_2_PO_4_, 6 mM MgCl_2_, 27 mM β-mercaptoethanol, 0.5% Triton X-100) and incubated at 37°C for 10 min. After 150 µL of the resulting lysate was added to 50 µL of p-nitrophenyl-β-D-galactopyranoside (PNPG) solution (0.9 mg/ml PNPG, 81 mM Na_2_HPO_4,_ 19 mM NaH_2_PO_4_) in a 96 well microplate, absorbance was measured at 405 nm every 5 min for 30 min at room temperature by a microplate reader (Molecular Devices’ Spectramax M2).

#### Yeast two-hybrid assay

A yeast two-hybrid assay was established by inserting *dprA* (prey) into yeast plasmid pGBDUC2 (containing DNA binding domain of GAL4 transcription factor), and inserting *comD*, *comE*, *comX*, *comW*, and *recA* (baits) into yeast plasmid pACT2 (containing transcription-activating domain of GAL4), using PCR followed by restriction enzyme digestion and ligation at BamHI and XhoI, or SalI sites. The two plasmids with corresponding inserts were first transformed into *E. coli* (DH5α) to replicate and purified with QIAprep Miniprep kit. The purified plasmid DNA was then transformed into yeast haploids PNS468 (MATE-a) and PNS752 (MATE-α) respectively. The two transformed haploid cells with different mating types were selected in SD medium without leucine (SD-Leu) or SD medium without uracil (SD-Ura) and mated to make diploids in SD agar plate without both (SD-Leu-Ura). The diploids were collected and stored at −80°C in 20% glycerol stocks. Diploids were grown again on YPD agar plates and frog-replicated onto four test agar plates: SD-Leu-Ura; SD-Leu-Ura-His; SD-Leu-Ura-His+1 mM 3AT; SD-Leu-Ura-His+3 mM 3AT [28]. The four test plates were photographed daily during incubation at 26°C for 7 days.

## Results

### Among Candidate Late Competence Genes Examined, Only *dprA* is Required for Normal Shutoff of Late Gene Expression in a Wild-type Background

To investigate the roles of additional late genes in competence termination, we monitored the expression pattern of a late gene transcriptional reporter, which is established as a reliable indicator of competence development [17]. For this purpose, we constructed a parent strain that contains a *lacZ* reporter at the late gene, *ssbB* and is also *comA* deficient, so as to avoid any potential growth deficiency that might be caused by prolonged competence in a mutant deficient in exit from competence. The late gene expression pattern in response to CSP in this reporter parent strain was verified as identical to that reported previously [7], [9], [16], [29]. Mutations in genes with known roles in transformation were crossed into the parent strain. Included were *cibABC* and *cbpD,* which affect fratricide, and *cinA,* linked to the cinbox promoter required for induction of *recA* by CSP. The internal deletion of each gene or operon was replaced by a Kn^R^ marker. Altogether, we examined 12 mutants, defective in a total of about 20 late genes (circled with in blue ovals in Fig. 1): *ΔcbpD, ΔcibABC, ΔssbB, ΔcglEFG, ΔcoiA, ΔdprA, ΔcelAB, ΔcclA, ΔcglABCD, ΔcflAB, ΔradA* and *ΔPc-cinA.* Each mutation’s structure was confirmed by PCR, and the border of each deletion is shown in Fig. 1. Since the transformation efficiency of these mutants was already known to be very low, we verified that transformation rates for the new strains were comparable to the values expected from the literature (Table 4).

**Table 4:** Comparison of transformation efficiency of new strains with literature.

New allelecombination		Relative transformation rate		
Strain(s)	Mutation	Literature^b^	Experimental^a^	
			ClpP^+^	ClpP^−^
*CP1250, CP2000*	*Δcps*	1		
*CPM7*	*ssbB^−^::lacZ::ssbB^+^*	1		
*CP1359*	*ΔclpP::PcTet*	1		
*CP2108, CP2125*	*ΔcomA::PcErm*	1	1	1
*CP2109, CP2126*	*ΔcbpD::PcKan*	1	1	1
*CP2110, CP2127*	*ΔcibABC::PcKan*	1	1	.4
*CP2111, CP2130*	*ΔcoiA::PcKan*	.01	.001	.001
*CP2112, CP2128*	*ΔcglEFG::PcKan*	0	.001	.001
*CP2113, CP2129*	*ΔdprA::PcKan*	0	.0001	.001
*CP2116, CP2131*	*ΔcelAB::PcKan*	0	.001	.001
*CP2114, CP2132*	*ΔcclA::PcKan*	0	.001	.0001
*CP2117, CP2133*	*ΔcglABCD::PcKan*	0	.0001	.0001
*CP2115, CP2134*	*ΔcflAB::PcKan*	0	.0001	.0001
*CP2119, CP2135*	*ΔradA::PcSpc*	0	.001	.001
*CP2139, CP2140*	*ΔssbB::PcKan*	.3	.3	0.5
CP2143, CP2144	Äcinbox-Ä*cinA*::PcKan	1	1	1

a Relative transformation rates were calculated by comparing the transformation efficiencies of the mutants to that of CP2000.

b The literature sources for the relative transformation rates of the mutants are found in Table 1 and/or in Table S2 in [7].

**Figure 2:**
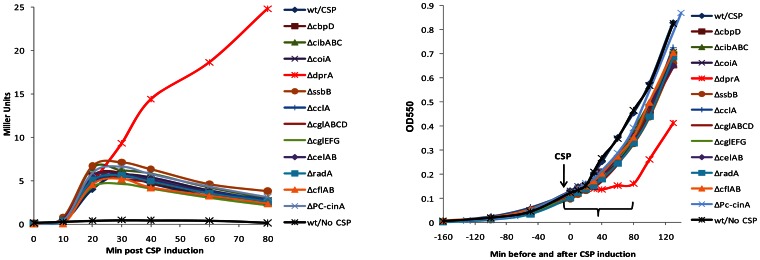
Survey of effect of late gene mutations on exit from the competent state. **A.** Late gene expression patterns were monitored in wild type and late gene mutants after CSP induction using a *lacZ* reporter inserted at the late gene *ssbB*. β-galactosidase activity (Miller units) was measured in culture samples harvested at indicated times after CSP induction. Each strain is indicated by its mutated competence gene (see Table 1). **B.** Growth patterns monitored as culture optical density at 550 nm before and after CSP treatment. Bracket indicates period of LacZ assay shown in panel **A**.

To determine the effect of each of the 12 mutations on exit from competence, each mutant reporter strain was treated with CSP under standard conditions and sampled 0, 10, 20, 30, 40, 60, or 80 minutes later for LacZ assay. The enzyme levels, which indicate late gene expression patterns, are shown as a function of the time after CSP induction in Fig. 2. Nearly all of these mutants displayed normal (brief) patterns of late gene expression, with a burst of LacZ synthesis restricted to the period between 10 and 30 min after addition of CSP. The single exception was the *dprA* mutant, in which late gene expression continued beyond 60 min The pattern shown in Fig. 2 reproduces the growth defect reported by Bergé [17] for the R6 strain, and further supports his interpretation by showing directly that expression of a late gene continues for an unusually long time after exposure of a *dprA* mutant to CSP; that is, the cells appear to remain in an active X-state for a greatly extended period when DprA is missing.

The greatly prolonged expression of a late gene suggests a prolonged presence of ComX and thus that DprA affects not only expression of the *comCDE* operon, but also expression of the duplicate *comX* genes. Combined with the report of Mirouze [18] that premature expression of *dprA* repairs the transience defect of a *comX* mutant, these results suggest that DprA is both necessary and sufficient for the shutoff of early gene expression. From the absence of any similar effect of the other late gene mutations on the temporal pattern of *ssbB* expression we conclude further that none of the other late gene products examined has a strong role in shutoff of early gene expression. Because of the uniquely strong effect of DprA on shutoff, it is of interest to determine its effects on gene expression in more detail, both to know if it is the only shutoff agent, and to identify its regulatory targets.

### The Kinetics of Exit from Competence is not Altered in a ClpP Protease-deficient Background

We were concerned to be able to detect regulation of *comX* at multiple levels. While the effect of DprA shown above is dramatic, as expected for a factor controlling early gene expression, the change in late gene expression kinetics might be more subtle for mutation of a gene reducing ComX activity directly. Thus, for all late gene mutants tested above, except the Δ*dprA* mutant, levels of ComX and ComW would be expected to start to decline by ∼30 minutes after CSP induction, simply due to the rapid *dprA*-dependent halt to early gene expression and subsequent decay of ComX and ComW that had accumulated during the brief window of ComE activity. Therefore, any extension of late gene expression occasioned by mutation of a gene acting specifically to suppress ComX activity during this window might be modest in length and difficult to detect. To extend the window of ComX availability and thus improve the chance of detecting such a ComX-dependent shutoff gene targeting ComX itself, we decided to study the temporal pattern of late gene expression in late gene mutants in a protease-deficient background (*ΔclpP*), in which both ComX and ComW would be stabilized. ClpP and ClpE are largely responsible for the proteolysis of ComX and in strains deficient for either of the two proteins, ComX becomes stable [30]. Similarly, ClpP and ClpC are largely responsible for the proteolysis of ComW and in strains deficient for one or the other protease subunit, ComW is stable [12].

**Figure 3:**
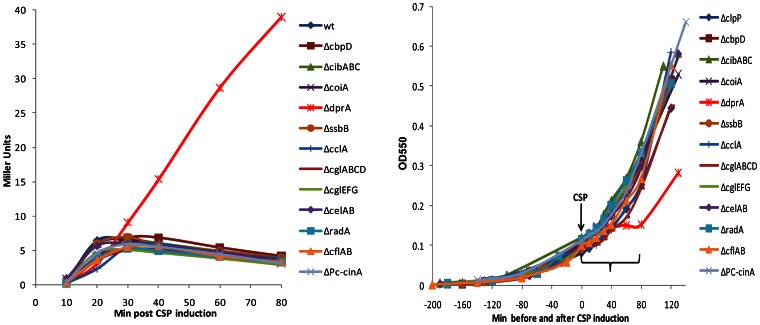
Survey of effect of late gene mutations on exit from the competent state in the protease deficient *ΔclpP* background. **A.** After CSP induction, late gene expression patterns were monitored in wild type and late gene mutants in *ΔclpP* background. The *lacZ* reporter and the measurement of β-galactosidase activity were done as described for Figure 2. Each strain is indicated by its mutant competence gene as listed in Table 1. **B.** Growth patterns monitored as culture optical density at 550 nm before and after CSP treatment. Bracket indicates period of LacZ assay shown in panel **A.**

Adopting the same strategy as in the previous section, a new parent strain, CP2125, was made from the original one by disruption of the *clpP* gene, to increase stability of both ComW and ComX proteins. From this new parent strain, we again obtained 12 late gene mutants: *ΔcbpD, ΔcibABC, ΔssbB, ΔcglEFG, ΔcoiA, ΔdprA, ΔcelAB, ΔcclA, ΔcglABCD, ΔcflAB, ΔradA* and *ΔPc-cinA,* and verified their structures and competence phenotypes as above. These mutants were analyzed for their late gene expression pattern after induction by CSP. In the *ΔclpP* background, we observed the same result as in the protease-proficient wild type: only the *dprA* mutant displayed prolonged late gene expression (with an elevated β-galactosidase activity, which may be due to the deficiency in host Clp proteases (Fig. 3). Since our survey of ∼19 late genes other than *dprA* did not reveal any whose loss extends the X-state when the half-lives of ComX and ComW are prolonged by interruption of their proteolysis, we conclude that none of these gene products is individually responsible for suppression of ComX activity during exit from competence.

### Accumulation of the Early Gene Products, ComX and ComW, is Increased and Prolonged in a *dprA* Mutant

**Figure 4:**
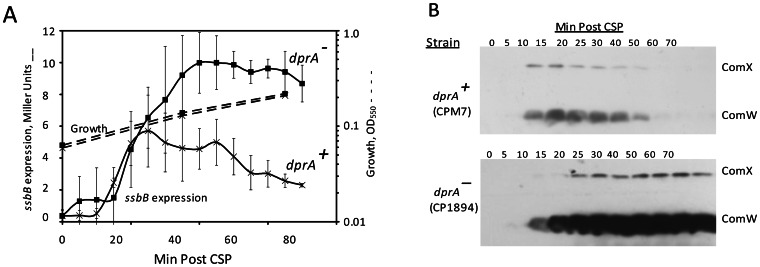
Prolonged appearance of ComX and ComW in a *dprA* mutant. **A.** Late gene expresion in strains CPM7 (x, *dprA* +) and CP1894 (□, *ΔdprA*) was monitored by measuring β-galactosidase activity (Miller units) (−) in a culture treated with CSP, using a *lacZ* transcriptional fusion to the *ssbB* promoter. Points are averages of three samples and error bars represent their standard deviation. –, growth of parallel non-CSP treated cultures. **B.** Western analysis of samples taken in parallel to samples for late gene expression run in a separate gel, transferred to a separate membrane for each strain and probed with antisera specific to both ComX and ComW using the ECL substrate and imaged on a separate film for each strain. Each lane represents a cell lysate from 0.6 ml of culture.

The prolonged late gene expression in a *ΔdprA* mutant might in principle reflect abrogation of a direct effect of DprA on late gene expression or interruption of an effect of DprA at the level of ComE, affecting early gene expression. These possibilities could be distinguished by comparing the accumulation of the early gene products, ComX and ComW, in a *dprA* mutant to that in wild type. If they displayed comparable levels of ComX and ComW, it would suggest action of DprA on late gene expression; whereas higher levels of ComX and ComW in the *dprA* strain would indicate that DprA shuts off expression of these additional early genes. To test the possibility that DprA negatively regulates the amounts of ComX and ComW produced during competence development, we used Western blotting to compare the levels of ComX and ComW in *dprA* **^+^** and *dprA* ^−^ strains upon treatment with CSP. As shown in Figure 4, both ComX and ComW accumulated too much higher levels in the *dprA* mutant and remained at a high level even after 70 min of exposure to CSP. In contrast, in the *dprA*+ strain ComX and ComW reached a lower maximum at 15–20 min, and then declined below detectable levels by 50 min. We conclude that *dprA* mutation prolonged competence by enhancing the accumulation of the early gene products, ComX and ComW.

### The Effect of DprA on the Accumulation of ComX and ComW does not Depend on ClpP

In light of the lability of ComX and ComW proteins, the difference in the CSP-induced levels of ComX and ComW between *dprA* ^−^ and *dprA* **^+^** strains might have two quite different explanations: 1) DprA may promote the proteolysis of ComX and ComW, acting as chaperon or adaptor that greatly stimulates the targeting of these proteins to the proteases responsible for their lability, ClpEP and ClpCP, respectively; or 2) DprA may inhibit the production of ComX and ComW, at either translational or transcriptional stages. To distinguish experimentally whether the increase in amounts of ComX and ComW caused by *dprA* mutation reflects a negative effect on proteolysis of the labile ComX and ComW proteins or, in parallel to the effect of DprA on expression of the *comCDE* operon, an enhancement of the rate of synthesis of these proteins, we compared the amounts of ComX and ComW in a *ΔclpPdprA^+^* mutant to the amounts in a *ΔclpP dprA* ^−^ mutant. As ClpP is necessary for the proteolysis of both ComX and ComW, both proteins are stable in a *ΔclpP* background and an anti-proteolytic mechanism would have no effect in that context.

**Figure 5:**
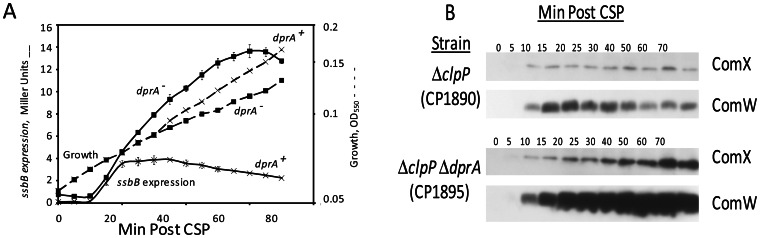
Prolonged accumulation of ComX and ComW in aΔ*clpPdprA* ^−^ mutant but not in a Δ*clpP* mutant. **A.** Late gene expression in strains CP1890 (x, *dprA*+ *ΔclpP*) and CP1895 (□, *ΔdprA ΔclpP*) was monitored by measuring β-galactosidase activity (Miller units) (–) in a culture treated with CSP. The *lacZ* reporter was in a transcriptional fusion to the *ssbB* promoter. Points are averages of three samples and error bars represent their standard deviation. –, growth of the cultures. **B.** Western blot analysis of samples taken in parallel to samples for late gene expression run in a separate gel, transferred to a separate membrane for each strain and probed with antisera specific to both ComX and ComW using the ECL substrate and imaged on a separate film for each strain. Each lane represents a cell lysate from 0.6 ml of culture.

Late gene expression differed radically between the two strains was (Figure 5), consistent with the result in Figure 4. The amounts of ComX and ComW, as determined by Western blot, continued to increase for more than 50 min in the *dprA clpP* mutant, whereas the levels of the two proteins became constant after 15 min in the *dprA*+ *clpP* mutant strain. The continued accumulation of both proteins well after the time that transcription of early genes would stop in a wild type strain [7] shows that DprA acts to block transcription (or translation) of both genes, not to promote proteolysis of these labile early proteins. Since ComX and ComW belong to distinct operons in the early class, and DprA suppresses transcription of the early operon *comCDE* [17], this now shows that three early operons respond similarly to deprivation of DprA. We conclude that DprA suppresses the transcription of at least three, and likely all, early genes.

### DprA Interacts with ComE

**Figure 6:**
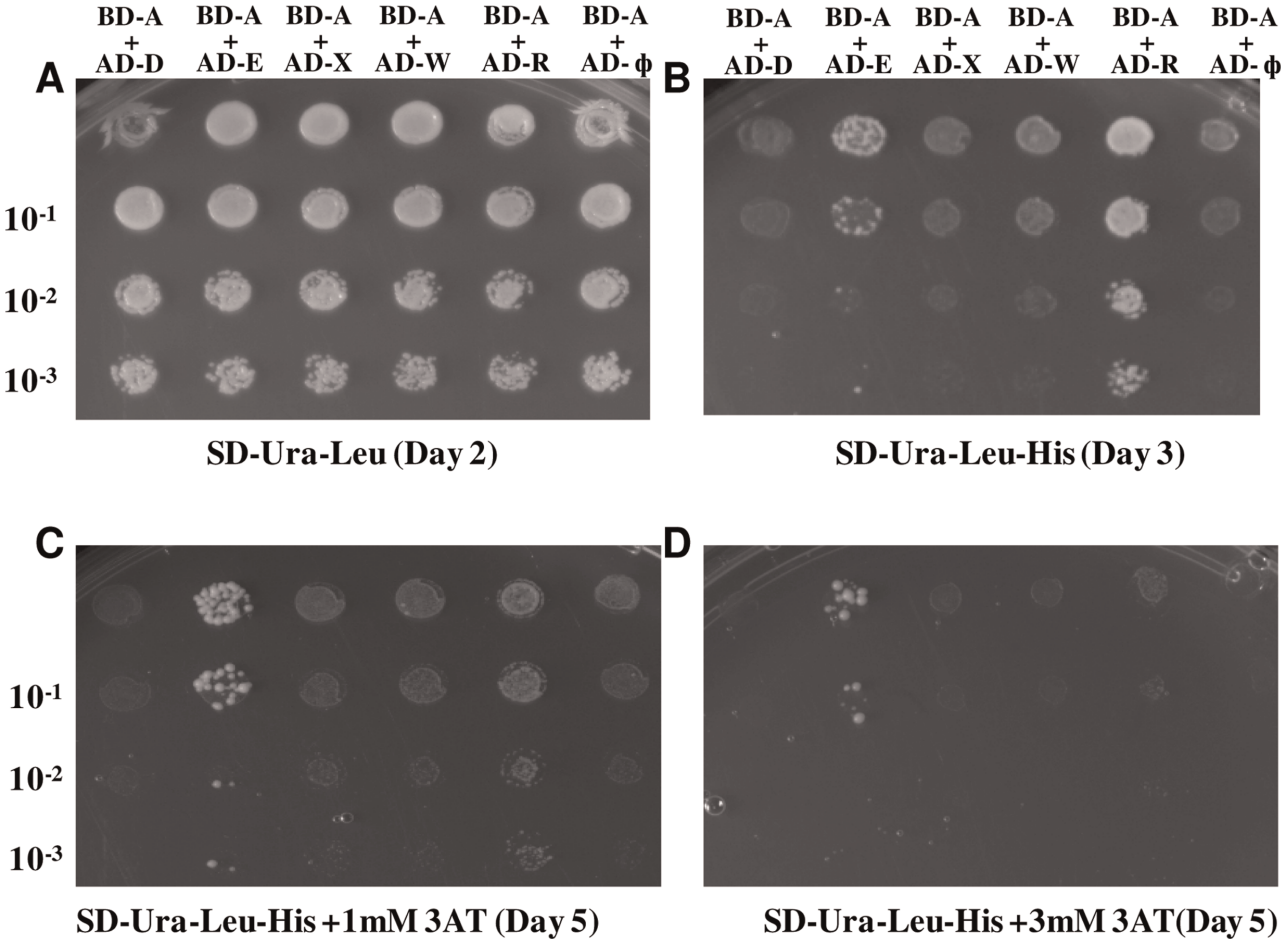
Screening of candidate DprA targets with yeast two-hybrid assay. Images were taken at indicated time during the incubation of the diploids (BD-A+AD-D, BD-A+AD-E, BD-A+AD-X, BD-A+AD-W, BD-A+AD-R, BD-A+AD-<$>\raster(70%)="rg1"<$>) in four kinds of agar plates for five days: SD-Ura-Leu (**A**), SD-Ura-Leu-His (**B**), SD-Ura-Leu-His+1 mM 3AT (**C**) and SD-Ura-Leu-His+3 mM 3AT (**D**). AD, activating domain of *pACT2*; BD, DNA binding domain of *pGBDCU2*; A, *dprA*; D, *comD*; E, *comE*; X, *comX*; W, *comW*; R, *recA*; SD, synthetic defined medium; Ura, uracil; Leu, leucine; His, histidine; 3AT, 3-Amino-1,2,4-triazole.

In view of the effect of DprA on early gene expression, we made a limited search to distinguish among possible molecular targets, through a yeast two-hybrid screen for protein interactions. ComD or ComE, the major players in turning on early gene expression, were the major candidates in our screen. Because DprA interacts with RecA [31], a *recA* insert was adopted as a positive control. Furthermore, because of results indicating that DprA does not affect late gene expression (described below), *comX* and *comW* inserts were included as negative controls. After confirmation of the constructions, *pGBDUC2* and *pGBDUC2*-*dprA* were transformed into yeast haploid NSY752(α). On the other hand, *pACT2*, *pACT2*-*comD*, *pACT2*-*comE*, *pACT2*-*comX*, *pACT2*-*comW* and *pACT2*-*recA* were transformed into yeast haploid NSY468(a). Six diploids were obtained by mating between NSY752(α) and NSY468(a) cell lines, as indicated in Figure 6.

After incubation of the diploids in four kinds of media (SD-Ura-Leu, SD-Ura-Leu-His, SD-Ura-Leu-His+1 mM 3AT and SD-Ura-Leu-His+3 mM 3AT), all diploids grew on SD-Ura-Leu (Figure 6A), confirming that plasmids pGBDUC2 and pACT2 (or their derivatives) were harbored respectively in the diploids. But in the plates without histidine (SD-Ura-Leu-His), only the diploids containing inserts *dprA*+*comE* and *dprA*+*recA* produced colonies. This indicates that DprA can interact with ComE as strongly as it interacts with RecA (Figure 6B). The growth in plates without histidine but with the competitive inhibitor of the production of histidine, 3AT, further confirmed the interaction between DprA and ComE is positive and strong, perhaps even stronger than the interaction between DprA and RecA because the diploid with *dprA* and *recA* inserts was unable to grow in the media SD-Ura-Leu-His+1 mM 3AT and SD-Ura-Leu-His+3 mM 3AT after a week incubation (Figure 6C, 6D). Further verification of the interaction between DprA and ComE was achieved with yeast two-hybrid by incorporating more negative controls, including double empty vectors and one empty vector with one vector plus insert (data not shown). Taken together, these results suggest a direct interaction between DprA and ComE, an interaction that might mediate its strong negative effect on expression of early genes.

### Late Gene Expression is Independent of DprA after Ectopic Induction of *comX* and *comW*

The prolonged transcription of the late gene reporter seen in *dprA* mutants contrasts with the rapid extinction of late gene expression in *clpP* mutants despite the continued presence of high levels of the otherwise labile ComX and ComW [12], suggesting that DprA or some other late *com* gene product might also play an additional regulatory role by affecting late gene transcription directly, supplementing any effects on early gene expression and the amount of ComX and ComW produced.

Since ComX and ComW are the only early gene products required for high levels of late gene transcription and cells can become fully competent when *comX* and *comW* are ectopically expressed under CSP-independent regulation [15], we sought to decouple late gene expression from early gene expression by introducing both *comX* and *comW* at the *aga* locus, to allow their expression under regulation by the raffinose-inducible *aga* promoter. By inducing competence development with raffinose while by-passing expression of the other early genes, DprA’s possible effect on late gene expression could thus be separated from its effect on early gene expression. The gene that codes for α-galactosidase, *aga*, was retained in the construct, so that expression from the Aga promoter could be monitored by measuring α-galactosidase activity [32], while the *ssbB*::*lacZ* reporter allowed monitoring of late gene expression by measuring β-galactosidase activity. To stabilize ComX and ComW, the new strain was also deficient in the ClpE and ClpC ATPases. The new strain, with ectopically regulated *comX* and *comW*, CP1902 (*aga::comX::comW*, Δ*clpC*, Δ*clpE*, *ssbB::lacZ::ssbB^+^*), was transformed with the *dprA* mutation of strain CP1389, to create the isogenic *dprA* derivative, strain CP1932.

**Figure 7:**
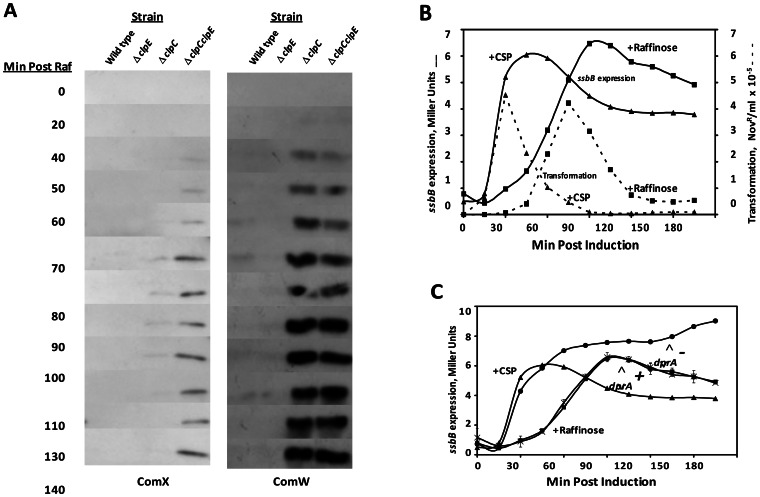
Lack of effect of DprA on late gene expression in an ectopic *comXcomW* strain. **A.** Western blot analysis of samples taken at different times after induction with raffinose. Wild type, CP1896 (*aga::comX::comW*); *ΔclpE*, CP1962 (CP1896, but *ΔclpE*); *ΔclpC*, CP1963 (CP1896, but *ΔclpC*); *ΔclpE ΔclpC,* CP1964 (CP1896, but *ΔclpE ΔclpC*). **B.** Kinetics of competence induction and late gene expression induced with either CSP (Δ) or raffinose (□). During treatment of CP1902 (*aga::comX::comW*, *ssbB::lacZ, ΔclpC*,*ΔclpE*,) with either CSP (250 ng/ml) or raffinose (0.1%) at 30°C, samples were taken in parallel, to monitor transformation (–) and α-gal activity (–). β-gal activity is expressed in Miller Units with respect to the OD of the culture at each time point. **C**. Late gene expression of CP1902 treated with either CSP (Δ) or raffinose (□) or CP1932 (CP1902, *ΔdprA*) with either CSP (•) or raffinose (x) at as 30°C for B.

Since the entire CSP sensing circuit is intact in both the ectopic *comX comW* strain CP1902 and the isogenic *dprA* derivative (CP1932), the two strains could be treated with CSP to induce competence development through the activity of early genes, or treated instead with raffinose, to induce competence without expression of any early genes other than *comX* and *comW*. When treated with CSP, the two different strains responded with different expression patterns, as expected, confirming the effect of the *dprA* mutation on the ‘early’ regulatory pathway (Fig. 7). When the two strains were treated instead with raffinose, the patterns of late gene expression in the *dprA* ^+^ and *dprA* ^−^ strains were identical (Fig. 7), except for a somewhat broader curve compared to CSP-induced late gene expression. The broader pattern reflects a slower rise in raffinose-induced ComX and ComW expression. Similar results were obtained with the *aga::comX nis::comW* strain constructed by Luo (data not shown). As *dprA* thus had no effect on late gene expression driven by ectopically expressed ComX and ComW, we conclude that DprA does not affect late gene expression by any direct effect on the activity of the alternative sigma factor, ComX. Instead, expression of the early genes via the CSP sensing circuit is necessary to reveal an effect of DprA on late gene expression. Logically, this also suggests that the difference in expression in the CSP treated *dprA* ^−^ vs *dprA* **^+^** cells is due solely to DprA’s effect on the regulation of early gene expression.

### CSP Induces Higher Levels of ComX and ComW in a dprA^−^ Background than does Raffinose in the Ectopic ComX/ComW Expression Regime

**Figure 8:**
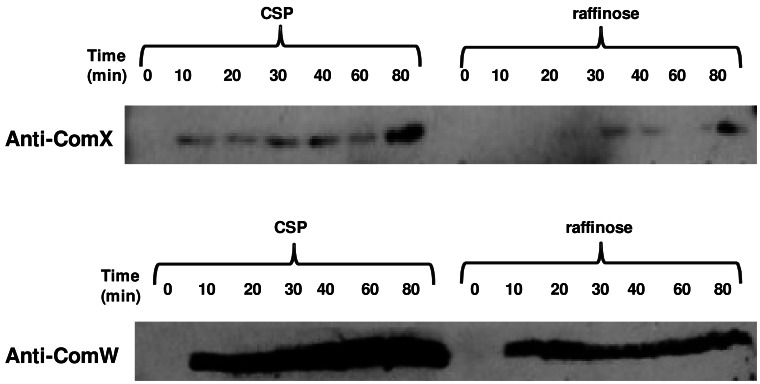
Reduced levels of ComX and ComW under ectopic regulation of competence. Late gene expression was induced in strain CP1932 (*aga::comX::comW*, *ssbB::lacZ,* Δ*clpC,* Δ*clpE* ) by 250 ng/ml CSP or 0.1% raffinose respectively at OD 0.1. The cultures were sampled at 0, 10, 20, 30, 40, 60, and 80 min after induction for western blotting assay. Two SDS-PAGE gels were run in parallel and probed with different antibodies: Top, PVDF membrane probed with anti-ComX antibody; Bottom, PVDF membrane probed with anti-ComW antibody.

Since late gene expression induced by raffinose was independent of a functional dprA gene, we infer that there may be another regulator, which reverses late gene expression soon after it begins. We further hypothesize that the apparent failure of this inhibition in the dprA mutant induced by CSP (Fig. 2 and Fig. 5) could be explained if ComX and ComW were produced in amounts far above those accumulated in the ectopically induced cells of Fig. 7. When ComX and ComW were produced in much higher amount, they might overwhelm the hypothetical regulator, without being affected by it.. To test this hypothesis, we compared levels of both ComX and ComW in CP1932 induced by CSP to the levels in the same strain induced by raffinose, sampling during 80 minutes after addition of the respective inducers. Two parallel SDS-gels were run, one probed with anti-ComX antibody and another probed with anti-ComW antibody. CSP-induced levels of both ComX and ComW were indeed much higher than those induced by raffinose (Figure 8), even while late gene expression, as represented by a ssbB reporter, though prolonged, was not increased in rate (Fig. 1, Fig. 7). Thus, the greatly elevated ratio of ComX to a hypothetical late gene product acting as an inhibitor could explain why late gene expression patterns in dprA mutants shut off after raffinose induction but not after induction by the native CSP pheromone (in Figure 7), and indicates the existence of another competence repressor.

## Discussion

The activity of alternative sigma factors is often controlled quite strictly at multiple levels, perhaps because they can cause a global shift in gene expression, which could be especially harmful if carried out under inappropriate circumstances. In the case of the alternative sigma factor central to competence development in S. pneumoniae, at least five distinct mechanisms of regulation are already established or glimpsed. (I) Transcription of comX depends on a TCSTS that coordinates within local populations and responds to unknown elicitors. (II) Production of ComX protein depends on an early competence gene, comW. (III) Separately, activity of ComX depends on ComW. (IV) ComX is labile, targeted by the ClpEClpP ATP-dependent protease. (V) ComW is labile, targeted by the ClpCClpP ATP-dependent protease. With the observations reported here on escape from the X state, two additional mechanisms that regulate the activity of ComX or ComW can be considered, both apparently forming negative feedback loops that ensure that induction of late gene expression is self-limiting and transient. One mechanism requires DprA and targets ComE, perhaps through direct interaction between these two proteins themselves; the other is less well defined, but is independent of dprA and appears to target ComX or other determinant of transcription of late genes.

Since ComX and ComW are central players during competence development and the disappearance of ComX and ComW occurs at about the same time as the loss of transformation [7], [9], [12], [14], it is attractive to suggest that the disappearance of ComX and ComW itself accounts for the shutoff of late gene expression [2], [3]. However, the relative timing of events during response to CSP is difficult to reconcile with this simple mechanism. Specifically, late gene mRNA largely disappears even before the levels of ComX and ComW proteins begin to drop, suggesting that the activity of ComX is itself subject to some additional form of control [6], [8], [12], [14]. As some non-proteolytic factor thus seems to play a major additional role in the shut off of ComX activity, we began to look for possible candidates among ComX-induced late genes. Peterson et al [7] screened mutations of many of the late genes for effects on the rate of exit from competence, but found none that caused a pronounced extension of the period of transformation. Transformation defective mutants were not examined for this phenotype, however, prompting us to investigate the latter class of late genes directly. dprA mutants treated with CSP displayed a prolonged period of expression of the late gene ssbB, in addition to the prolonged expression of the early gene operon comCDE previously described as a mutant phenotype for dprA by Bergé [17] and by Mirouze et al [18]. Comparison of levels of ComX and ComW in dprA mutants vs wild type in protease defective backgrounds revealed that DprA not only turns off expression of the early gene operon comCDE, but also has a parallel effect on the early genes comX and comW, thus strongly suggesting an effect on expression of all early genes. Our present results suggest that the target of DprA action within the regulators of early gene expression may be ComE itself. This would provide a direct path to permitting exit from the competent state, although less direct effects of DprA or of its complexes with RecA or with ssDNA cannot be ruled out. As a response regulator of a two component system, phosphorylated ComE accounts for turning on early gene expression. It is our speculation that DprA, via interacting with ComE, might cause de-phosphorylation of ComE, or hinder its recruitment of RNAP to promoter regions, to control early gene expression. Further studies, including distinguishing the two mechanisms and identifying interacting surfaces in both proteins, are warranted.

Recently published results of a parallel study establishing a key role for DprA in exit from competence are based on comX-independent exit from competence when dprA is converted essentially into an early gene [33]. The present study, based on the complementary approach of removal of individual late genes to reveal which is needed for comX-dependent exit from competence, strengthens this conclusion.The apparent role of DprA in terminating late gene transcription could thus in principle be either a secondary effect of its inhibition of early gene expression or could reflect an additional direct effect on late gene expression. To see if DprA also restricted late gene expression directly by an effect on ComX, a new strain was created in this study for the ectopic expression of comX and comW. This new strain can develop competence upon induction of comX and comW by raffinose treatment. In this strain, the ability to transform was transient despite continued presence of ComX and ComW (Fig. 7), providing a good background to evaluate the possibility of a direct effect of DprA on late gene expression. In a dprA mutant derivative of this strain, the pattern of late gene expression following comX and comW induction perfectly matched the pattern in the dprA**^+^** parent. This strongly suggests that DprA does not suppress ComX activity in late gene transcription, but that another gene may be responsible for limiting ComX activity to a short time window. This hypothetic regulator is unlikely to be CSP-induced because it could be induced by raffinose, but instead appears to be dependent, directly or indirectly, on induction of comX (or comW),

It remains a challenge to reconcile the different patterns of late gene expression in *dprA* mutants under CSP and raffinose inductions. Specifically, why is late gene expression prolonged in the *dprA* mutant if there is a separate (late) inhibitor directly targeting ComX? We propose that DprA is not the only factor that shuts off competence, but that a second independent inhibitor accounts for the termination of late gene expression in *dprA* mutants in the ectopic expression system as well as for the prompt termination of late gene transcription in the WT while ComX is still present [12], [14]. As this second repressor was apparently ineffective in CSP-induced cultures in the *dprA* mutant background, we hypothesize that ComX and ComW are accumulated to different levels with CSP and raffinose. In the Δ*dprA* mutant induced by CSP, where DprA, the factor normally curbing early gene expression, is removed, there would be a continuous supply of ComX and ComW at elevated levels, so that even if the second repressor could inactivate a normal amount of ComX or ComW, it could be overwhelmed by the unusually high amounts of these regulators. Direct comparison verified this inferred difference in the levels of ComX and ComW achieved under the two expression regimes, but the hypothetical factor responsible for the shut off of late gene expression remains unknown. While here we have ruled out ∼20 late gene products, several other late genes as well as the entire class of ‘delayed’ genes remain untested. Since competence in *S. pneumoniae* both imposes a stress on the competent cell itself and creates a potential hazard to nearby cells, it should not be surprising that its initiation and termination are both controlled at multiple levels.
